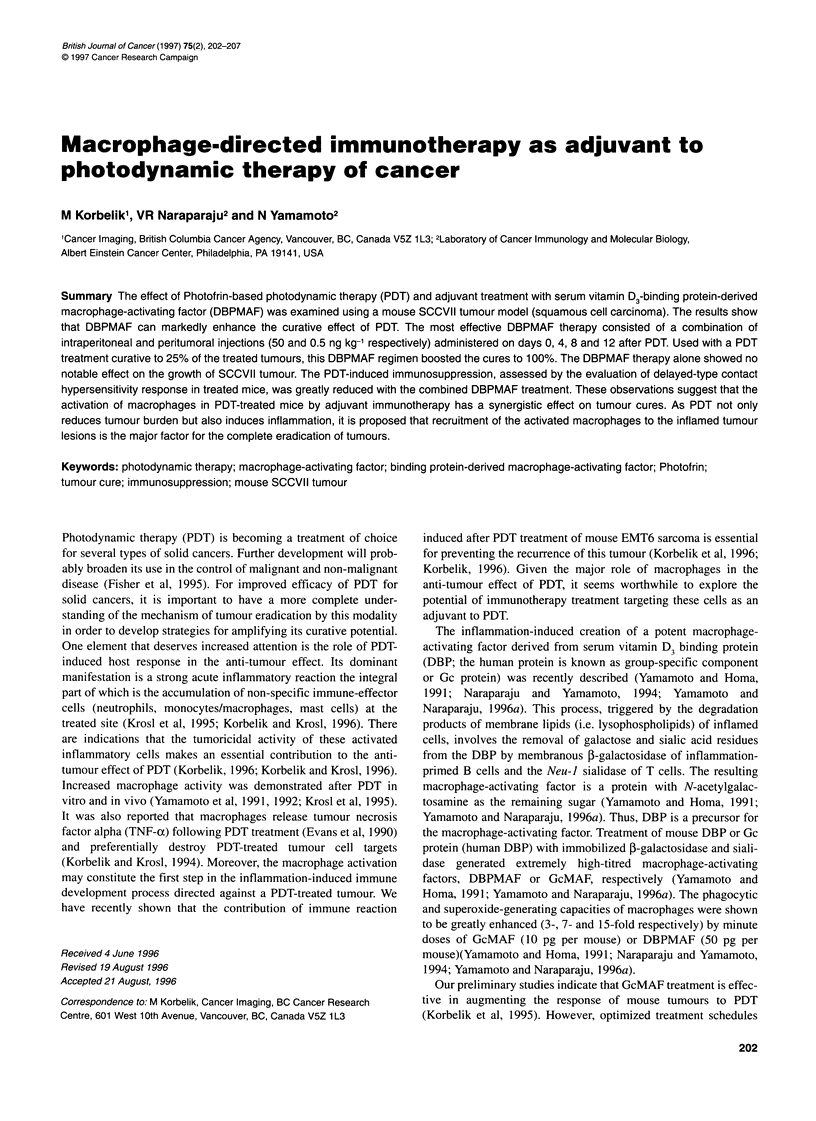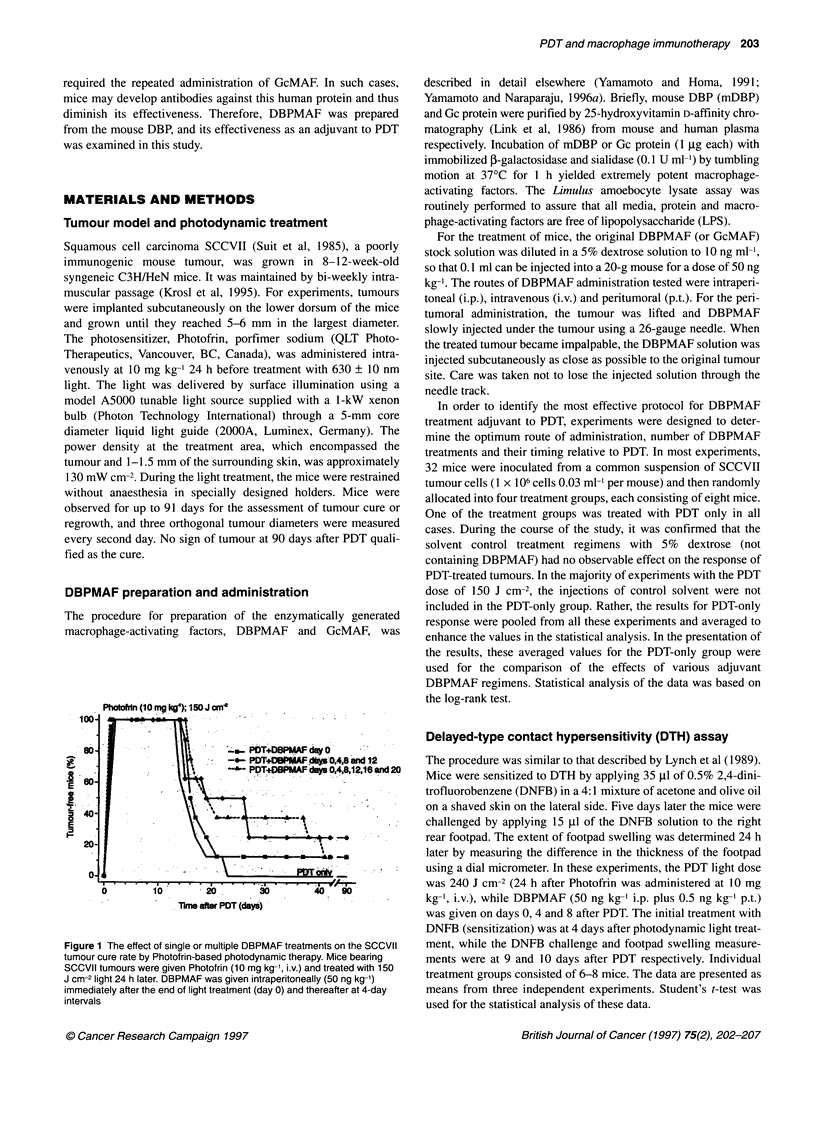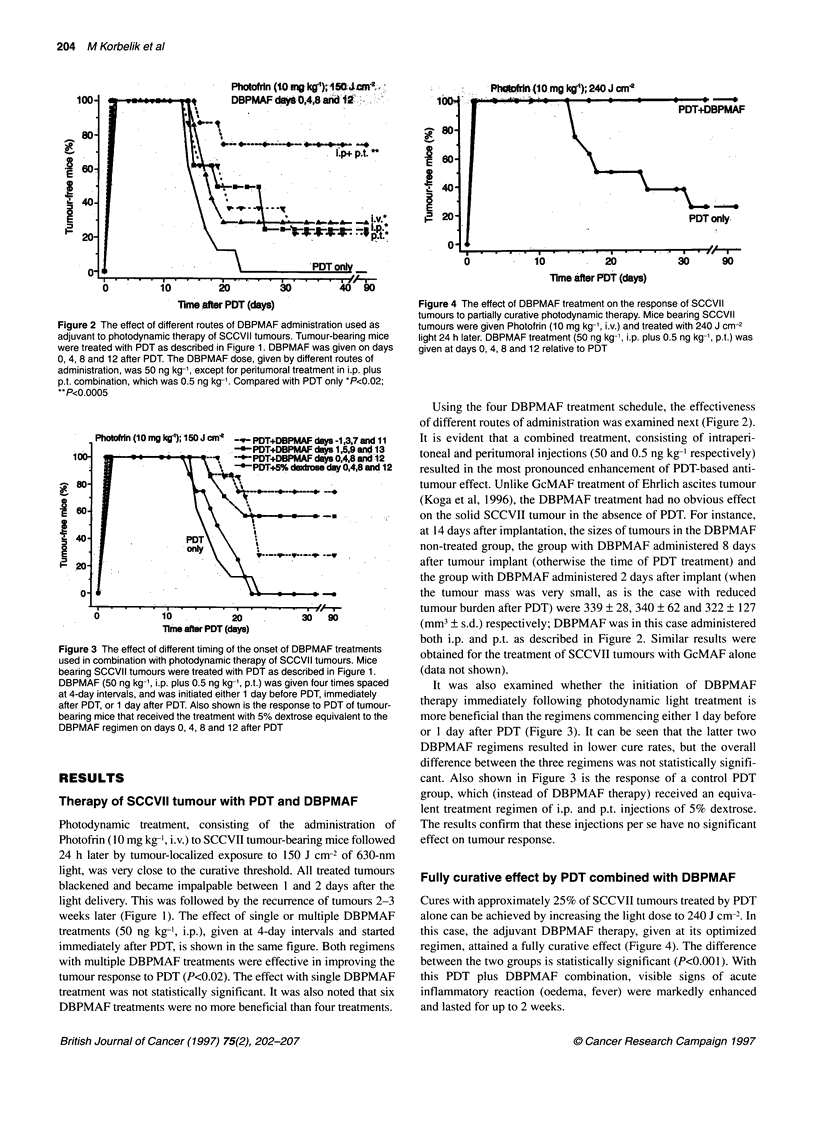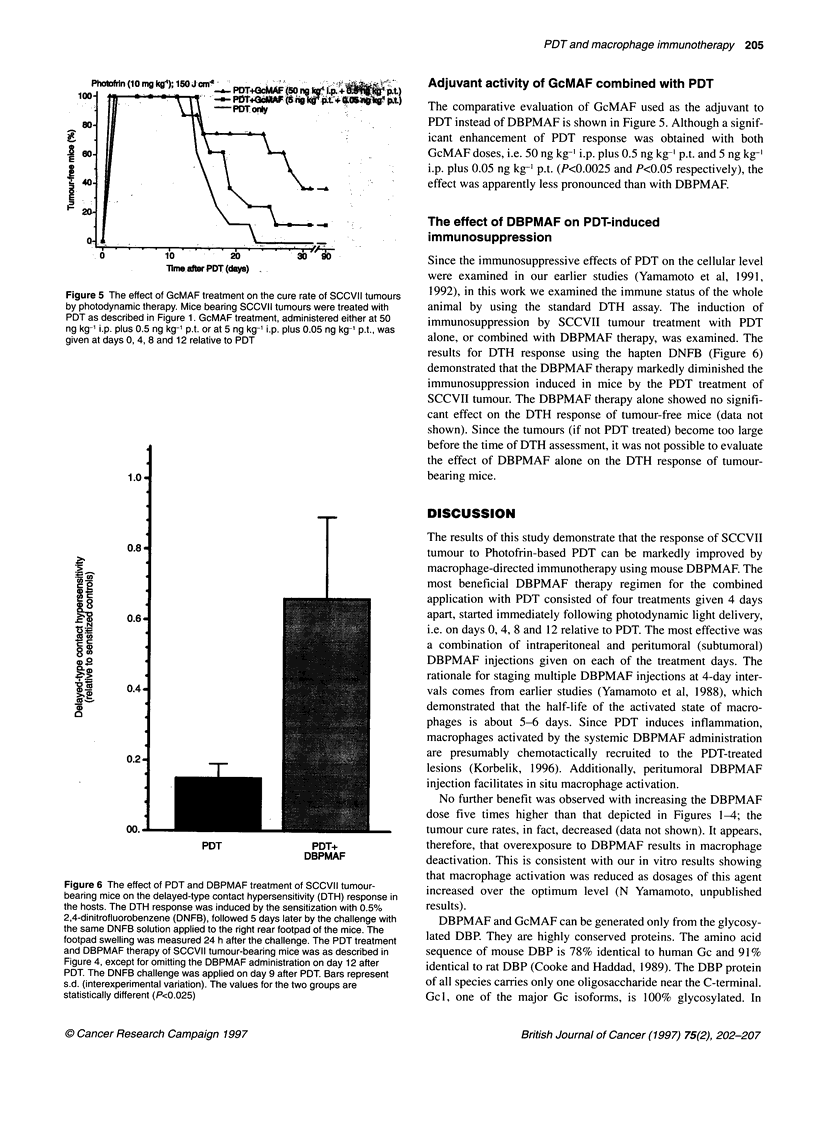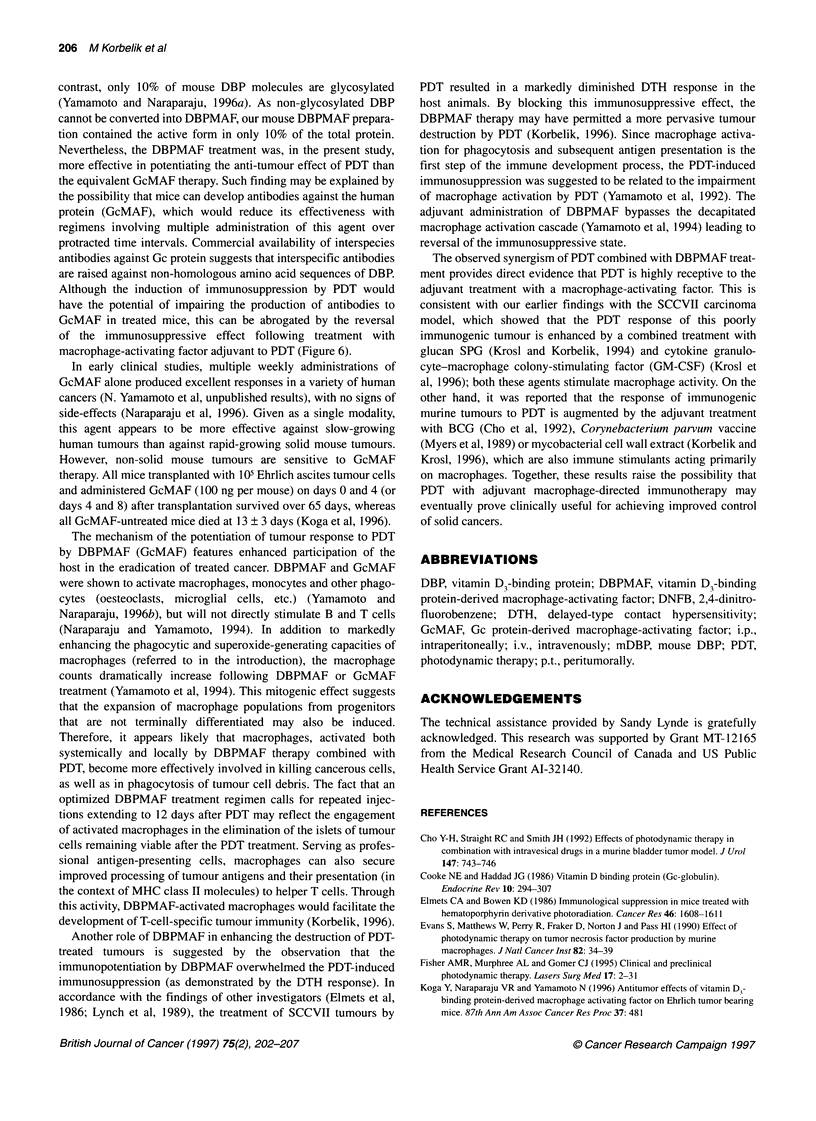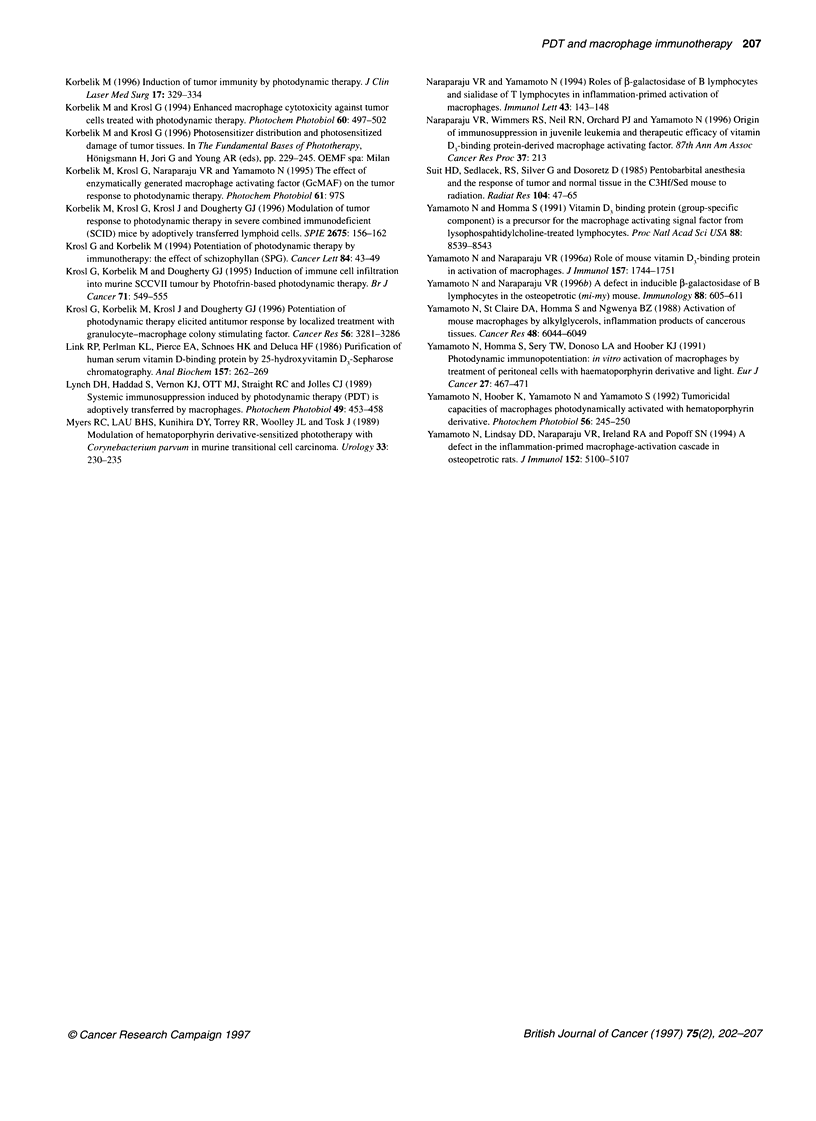# Macrophage-directed immunotherapy as adjuvant to photodynamic therapy of cancer.

**DOI:** 10.1038/bjc.1997.34

**Published:** 1997

**Authors:** M. Korbelik, V. R. Naraparaju, N. Yamamoto

**Affiliations:** Cancer Imaging, British Columbia Cancer Agency, Vancouver, Canada.

## Abstract

The effect of Photofrin-based photodynamic therapy (PDT) and adjuvant treatment with serum vitamin D3-binding protein-derived macrophage-activating factor (DBPMAF) was examined using a mouse SCCVII tumour model (squamous cell carcinoma). The results show that DBPMAF can markedly enhance the curative effect of PDT. The most effective DBPMAF therapy consisted of a combination of intraperitoneal and peritumoral injections (50 and 0.5 ng kg-1 respectively) administered on days 0, 4, 8 and 12 after PDT. Used with a PDT treatment curative to 25% of the treated tumours, this DBPMAF regimen boosted the cures to 100%. The DBPMAF therapy alone showed no notable effect on the growth of SCCVII tumour. The PDT-induced immunosuppression, assessed by the evaluation of delayed-type contact hypersensitivity response in treated mice, was greatly reduced with the combined DBPMAF treatment. These observations suggest that the activation of macrophages in PDT-treated mice by adjuvant immunotherapy has a synergistic effect on tumour cures. As PDT not only reduces tumour burden but also induces inflammation, it is proposed that recruitment of the activated macrophages to the inflamed tumour lesions is the major factor for the complete eradication of tumours.


British Journal of Cancer (1997) 75(2), 202–207
© 1997 Cancer Research Campaign

### Macrophage-directed immunotherapy as adjuvant to photodynamic therapy of cancer

**M Korbelik[1], VR Naraparaju[2] and N Yamamoto[2]**

[1]Cancer Imaging, British Columbia Cancer Agency, Vancouver, BC, Canada V5Z 1L3; [2]Laboratory of Cancer Immunology and Molecular Biology, Albert Einstein Cancer Center, Philadelphia, PA 19141, USA

**Summary** The effect of Photofrin-based photodynamic therapy (PDT) and adjuvant treatment with serum vitamin $D_3$-binding protein-derived macrophage-activating factor (DBPMAF) was examined using a mouse SCCVII tumour model (squamous cell carcinoma). The results show that DBPMAF can markedly enhance the curative effect of PDT. The most effective DBPMAF therapy consisted of a combination of intraperitoneal and peritumoral injections (50 and 0.5 ng $kg^{-1}$ respectively) administered on days 0, 4, 8 and 12 after PDT. Used with a PDT treatment curative to 25% of the treated tumours, this DBPMAF regimen boosted the cures to 100%. The DBPMAF therapy alone showed no notable effect on the growth of SCCVII tumour. The PDT-induced immunosuppression, assessed by the evaluation of delayed-type contact hypersensitivity response in treated mice, was greatly reduced with the combined DBPMAF treatment. These observations suggest that the activation of macrophages in PDT-treated mice by adjuvant immunotherapy has a synergistic effect on tumour cures. As PDT not only reduces tumour burden but also induces inflammation, it is proposed that recruitment of the activated macrophages to the inflamed tumour lesions is the major factor for the complete eradication of tumours.

**Keywords:** photodynamic therapy; macrophage-activating factor; binding protein-derived macrophage-activating factor; Photofrin; tumour cure; immunosuppression; mouse SCCVII tumour

Photodynamic therapy (PDT) is becoming a treatment of choice for several types of solid cancers. Further development will probably broaden its use in the control of malignant and non-malignant disease (Fisher et al, 1995). For improved efficacy of PDT for solid cancers, it is important to have a more complete understanding of the mechanism of tumour eradication by this modality in order to develop strategies for amplifying its curative potential. One element that deserves increased attention is the role of PDT-induced host response in the anti-tumour effect. Its dominant manifestation is a strong acute inflammatory reaction the integral part of which is the accumulation of non-specific immune-effector cells (neutrophils, monocytes/macrophages, mast cells) at the treated site (Krosl et al, 1995; Korbelik and Krosl, 1996). There are indications that the tumoricidal activity of these activated inflammatory cells makes an essential contribution to the anti-tumour effect of PDT (Korbelik, 1996; Korbelik and Krosl, 1996). Increased macrophage activity was demonstrated after PDT in vitro and in vivo (Yamamoto et al, 1991, 1992; Krosl et al, 1995). It was also reported that macrophages release tumour necrosis factor alpha (TNF-$\alpha$) following PDT treatment (Evans et al, 1990) and preferentially destroy PDT-treated tumour cell targets (Korbelik and Krosl, 1994). Moreover, the macrophage activation may constitute the first step in the inflammation-induced immune development process directed against a PDT-treated tumour. We have recently shown that the contribution of immune reaction induced after PDT treatment of mouse EMT6 sarcoma is essential for preventing the recurrence of this tumour (Korbelik et al, 1996; Korbelik, 1996). Given the major role of macrophages in the anti-tumour effect of PDT, it seems worthwhile to explore the potential of immunotherapy treatment targeting these cells as an adjuvant to PDT.

The inflammation-induced creation of a potent macrophage-activating factor derived from serum vitamin $D_3$ binding protein (DBP; the human protein is known as group-specific component or Gc protein) was recently described (Yamamoto and Homa, 1991; Naraparaju and Yamamoto, 1994; Yamamoto and Naraparaju, 1996*a*). This process, triggered by the degradation products of membrane lipids (i.e. lysophospholipids) of inflamed cells, involves the removal of galactose and sialic acid residues from the DBP by membranous $\beta$-galactosidase of inflammation-primed B cells and the *Neu-1* sialidase of T cells. The resulting macrophage-activating factor is a protein with *N*-acetylgalactosamine as the remaining sugar (Yamamoto and Homa, 1991; Yamamoto and Naraparaju, 1996*a*). Thus, DBP is a precursor for the macrophage-activating factor. Treatment of mouse DBP or Gc protein (human DBP) with immobilized $\beta$-galactosidase and sialidase generated extremely high-titred macrophage-activating factors, DBPMAF or GcMAF, respectively (Yamamoto and Homa, 1991; Yamamoto and Naraparaju, 1996*a*). The phagocytic and superoxide-generating capacities of macrophages were shown to be greatly enhanced (3-, 7- and 15-fold respectively) by minute doses of GcMAF (10 pg per mouse) or DBPMAF (50 pg per mouse)(Yamamoto and Homa, 1991; Naraparaju and Yamamoto, 1994; Yamamoto and Naraparaju, 1996*a*).

Our preliminary studies indicate that GcMAF treatment is effective in augmenting the response of mouse tumours to PDT (Korbelik et al, 1995). However, optimized treatment schedules

*Received 4 June 1996*
*Revised 19 August 1996*
*Accepted 21 August, 1996*

*Correspondence to:* M Korbelik, Cancer Imaging, BC Cancer Research Centre, 601 West 10th Avenue, Vancouver, BC, Canada V5Z 1L3

required the repeated administration of GcMAF. In such cases, mice may develop antibodies against this human protein and thus diminish its effectiveness. Therefore, DBPMAF was prepared from the mouse DBP, and its effectiveness as an adjuvant to PDT was examined in this study.

### MATERIALS AND METHODS

### Tumour model and photodynamic treatment

Squamous cell carcinoma SCCVII (Suit et al, 1985), a poorly immunogenic mouse tumour, was grown in 8–12-week-old syngeneic C3H/HeN mice. It was maintained by bi-weekly intramuscular passage (Krosl et al, 1995). For experiments, tumours were implanted subcutaneously on the lower dorsum of the mice and grown until they reached 5–6 mm in the largest diameter. The photosensitizer, Photofrin, porfimer sodium (QLT PhotoTherapeutics, Vancouver, BC, Canada), was administered intravenously at 10 mg $kg^{-1}$ 24 h before treatment with 630 ± 10 nm light. The light was delivered by surface illumination using a model A5000 tunable light source supplied with a 1-kW xenon bulb (Photon Technology International) through a 5-mm core diameter liquid light guide (2000A, Luminex, Germany). The power density at the treatment area, which encompassed the tumour and 1–1.5 mm of the surrounding skin, was approximately 130 mW $cm^{-2}$. During the light treatment, the mice were restrained without anaesthesia in specially designed holders. Mice were observed for up to 91 days for the assessment of tumour cure or regrowth, and three orthogonal tumour diameters were measured every second day. No sign of tumour at 90 days after PDT qualified as the cure.

### DBPMAF preparation and administration

The procedure for preparation of the enzymatically generated macrophage-activating factors, DBPMAF and GcMAF, was described in detail elsewhere (Yamamoto and Homa, 1991; Yamamoto and Naraparaju, 1996*a*). Briefly, mouse DBP (mDBP) and Gc protein were purified by 25-hydroxyvitamin D-affinity chromatography (Link et al, 1986) from mouse and human plasma respectively. Incubation of mDBP or Gc protein (1 μg each) with immobilized β-galactosidase and sialidase (0.1 U $ml^{-1}$) by tumbling motion at 37°C for 1 h yielded extremely potent macrophage-activating factors. The *Limulus* amoebocyte lysate assay was routinely performed to assure that all media, protein and macrophage-activating factors are free of lipopolysaccharide (LPS).

For the treatment of mice, the original DBPMAF (or GcMAF) stock solution was diluted in a 5% dextrose solution to 10 ng $ml^{-1}$, so that 0.1 ml can be injected into a 20-g mouse for a dose of 50 ng $kg^{-1}$. The routes of DBPMAF administration tested were intraperitoneal (i.p.), intravenous (i.v.) and peritumoral (p.t.). For the peritumoral administration, the tumour was lifted and DBPMAF slowly injected under the tumour using a 26-gauge needle. When the treated tumour became impalpable, the DBPMAF solution was injected subcutaneously as close as possible to the original tumour site. Care was taken not to lose the injected solution through the needle track.

In order to identify the most effective protocol for DBPMAF treatment adjuvant to PDT, experiments were designed to determine the optimum route of administration, number of DBPMAF treatments and their timing relative to PDT. In most experiments, 32 mice were inoculated from a common suspension of SCCVII tumour cells (1 × $10^6$ cells 0.03 $ml^{-1}$ per mouse) and then randomly allocated into four treatment groups, each consisting of eight mice. One of the treatment groups was treated with PDT only in all cases. During the course of the study, it was confirmed that the solvent control treatment regimens with 5% dextrose (not containing DBPMAF) had no observable effect on the response of PDT-treated tumours. In the majority of experiments with the PDT dose of 150 J $cm^{-2}$, the injections of control solvent were not included in the PDT-only group. Rather, the results for PDT-only response were pooled from all these experiments and averaged to enhance the values in the statistical analysis. In the presentation of the results, these averaged values for the PDT-only group were used for the comparison of the effects of various adjuvant DBPMAF regimens. Statistical analysis of the data was based on the log-rank test.

**Figure 1** The effect of single or multiple DBPMAF treatments on the SCCVII tumour cure rate by Photofrin-based photodynamic therapy. Mice bearing SCCVII tumours were given Photofrin (10 mg $kg^{-1}$, i.v.) and treated with 150 J $cm^{-2}$ light 24 h later. DBPMAF was given intraperitoneally (50 ng $kg^{-1}$) immediately after the end of light treatment (day 0) and thereafter at 4-day intervals

### Delayed-type contact hypersensitivity (DTH) assay

The procedure was similar to that described by Lynch et al (1989). Mice were sensitized to DTH by applying 35 μl of 0.5% 2,4-dinitrofluorobenzene (DNFB) in a 4:1 mixture of acetone and olive oil on a shaved skin on the lateral side. Five days later the mice were challenged by applying 15 μl of the DNFB solution to the right rear footpad. The extent of footpad swelling was determined 24 h later by measuring the difference in the thickness of the footpad using a dial micrometer. In these experiments, the PDT light dose was 240 J $cm^{-2}$ (24 h after Photofrin was administered at 10 mg $kg^{-1}$, i.v.), while DBPMAF (50 ng $kg^{-1}$ i.p. plus 0.5 ng $kg^{-1}$ p.t.) was given on days 0, 4 and 8 after PDT. The initial treatment with DNFB (sensitization) was at 4 days after photodynamic light treatment, while the DNFB challenge and footpad swelling measurements were at 9 and 10 days after PDT respectively. Individual treatment groups consisted of 6–8 mice. The data are presented as means from three independent experiments. Student's *t*-test was used for the statistical analysis of these data.

© Cancer Research Campaign 1997

**Figure 2** The effect of different routes of DBPMAF administration used as adjuvant to photodynamic therapy of SCCVII tumours. Tumour-bearing mice were treated with PDT as described in Figure 1. DBPMAF was given on days 0, 4, 8 and 12 after PDT. The DBPMAF dose, given by different routes of administration, was 50 ng kg$^{-1}$, except for peritumoral treatment in i.p. plus p.t. combination, which was 0.5 ng kg$^{-1}$. Compared with PDT only **P*<0.02; ***P*<0.0005

**Figure 3** The effect of different timing of the onset of DBPMAF treatments used in combination with photodynamic therapy of SCCVII tumours. Mice bearing SCCVII tumours were treated with PDT as described in Figure 1. DBPMAF (50 ng kg$^{-1}$, i.p. plus 0.5 ng kg$^{-1}$, p.t.) was given four times spaced at 4-day intervals, and was initiated either 1 day before PDT, immediately after PDT, or 1 day after PDT. Also shown is the response to PDT of tumour-bearing mice that received the treatment with 5% dextrose equivalent to the DBPMAF regimen on days 0, 4, 8 and 12 after PDT

### RESULTS

### Therapy of SCCVII tumour with PDT and DBPMAF

Photodynamic treatment, consisting of the administration of Photofrin (10 mg kg$^{-1}$, i.v.) to SCCVII tumour-bearing mice followed 24 h later by tumour-localized exposure to 150 J cm$^{-2}$ of 630-nm light, was very close to the curative threshold. All treated tumours blackened and became impalpable between 1 and 2 days after the light delivery. This was followed by the recurrence of tumours 2–3 weeks later (Figure 1). The effect of single or multiple DBPMAF treatments (50 ng kg$^{-1}$, i.p.), given at 4-day intervals and started immediately after PDT, is shown in the same figure. Both regimens with multiple DBPMAF treatments were effective in improving the tumour response to PDT ($P$<0.02). The effect with single DBPMAF treatment was not statistically significant. It was also noted that six DBPMAF treatments were no more beneficial than four treatments.

**Figure 4** The effect of DBPMAF treatment on the response of SCCVII tumours to partially curative photodynamic therapy. Mice bearing SCCVII tumours were given Photofrin (10 mg kg$^{-1}$, i.v.) and treated with 240 J cm$^{-2}$ light 24 h later. DBPMAF treatment (50 ng kg$^{-1}$, i.p. plus 0.5 ng kg$^{-1}$, p.t.) was given at days 0, 4, 8 and 12 relative to PDT

Using the four DBPMAF treatment schedule, the effectiveness of different routes of administration was examined next (Figure 2). It is evident that a combined treatment, consisting of intraperitoneal and peritumoral injections (50 and 0.5 ng kg$^{-1}$ respectively) resulted in the most pronounced enhancement of PDT-based antitumour effect. Unlike GcMAF treatment of Ehrlich ascites tumour (Koga et al, 1996), the DBPMAF treatment had no obvious effect on the solid SCCVII tumour in the absence of PDT. For instance, at 14 days after implantation, the sizes of tumours in the DBPMAF non-treated group, the group with DBPMAF administered 8 days after tumour implant (otherwise the time of PDT treatment) and the group with DBPMAF administered 2 days after implant (when the tumour mass was very small, as is the case with reduced tumour burden after PDT) were 339 ± 28, 340 ± 62 and 322 ± 127 (mm$^3$ ± s.d.) respectively; DBPMAF was in this case administered both i.p. and p.t. as described in Figure 2. Similar results were obtained for the treatment of SCCVII tumours with GcMAF alone (data not shown).

It was also examined whether the initiation of DBPMAF therapy immediately following photodynamic light treatment is more beneficial than the regimens commencing either 1 day before or 1 day after PDT (Figure 3). It can be seen that the latter two DBPMAF regimens resulted in lower cure rates, but the overall difference between the three regimens was not statistically significant. Also shown in Figure 3 is the response of a control PDT group, which (instead of DBPMAF therapy) received an equivalent treatment regimen of i.p. and p.t. injections of 5% dextrose. The results confirm that these injections per se have no significant effect on tumour response.

### Fully curative effect by PDT combined with DBPMAF

Cures with approximately 25% of SCCVII tumours treated by PDT alone can be achieved by increasing the light dose to 240 J cm$^{-2}$. In this case, the adjuvant DBPMAF therapy, given at its optimized regimen, attained a fully curative effect (Figure 4). The difference between the two groups is statistically significant ($P$<0.001). With this PDT plus DBPMAF combination, visible signs of acute inflammatory reaction (oedema, fever) were markedly enhanced and lasted for up to 2 weeks.

 © Cancer Research Campaign 1997

**Figure 5** The effect of GcMAF treatment on the cure rate of SCCVII tumours by photodynamic therapy. Mice bearing SCCVII tumours were treated with PDT as described in Figure 1. GcMAF treatment, administered either at 50 ng $kg^{-1}$ i.p. plus 0.5 ng $kg^{-1}$ p.t. or at 5 ng $kg^{-1}$ i.p. plus 0.05 ng $kg^{-1}$ p.t., was given at days 0, 4, 8 and 12 relative to PDT

**Figure 6** The effect of PDT and DBPMAF treatment of SCCVII tumour-bearing mice on the delayed-type contact hypersensitivity (DTH) response in the hosts. The DTH response was induced by the sensitization with 0.5% 2,4-dinitrofluorobenzene (DNFB), followed 5 days later by the challenge with the same DNFB solution applied to the right rear footpad of the mice. The footpad swelling was measured 24 h after the challenge. The PDT treatment and DBPMAF therapy of SCCVII tumour-bearing mice was as described in Figure 4, except for omitting the DBPMAF administration on day 12 after PDT. The DNFB challenge was applied on day 9 after PDT. Bars represent s.d. (interexperimental variation). The values for the two groups are statistically different ($P$<0.025)

### Adjuvant activity of GcMAF combined with PDT

The comparative evaluation of GcMAF used as the adjuvant to PDT instead of DBPMAF is shown in Figure 5. Although a significant enhancement of PDT response was obtained with both GcMAF doses, i.e. 50 ng $kg^{-1}$ i.p. plus 0.5 ng $kg^{-1}$ p.t. and 5 ng $kg^{-1}$ i.p. plus 0.05 ng $kg^{-1}$ p.t. ($P$<0.0025 and $P$<0.05 respectively), the effect was apparently less pronounced than with DBPMAF.

### The effect of DBPMAF on PDT-induced immunosuppression

Since the immunosuppressive effects of PDT on the cellular level were examined in our earlier studies (Yamamoto et al, 1991, 1992), in this work we examined the immune status of the whole animal by using the standard DTH assay. The induction of immunosuppression by SCCVII tumour treatment with PDT alone, or combined with DBPMAF therapy, was examined. The results for DTH response using the hapten DNFB (Figure 6) demonstrated that the DBPMAF therapy markedly diminished the immunosuppression induced in mice by the PDT treatment of SCCVII tumour. The DBPMAF therapy alone showed no significant effect on the DTH response of tumour-free mice (data not shown). Since the tumours (if not PDT treated) become too large before the time of DTH assessment, it was not possible to evaluate the effect of DBPMAF alone on the DTH response of tumour-bearing mice.

### DISCUSSION

The results of this study demonstrate that the response of SCCVII tumour to Photofrin-based PDT can be markedly improved by macrophage-directed immunotherapy using mouse DBPMAF. The most beneficial DBPMAF therapy regimen for the combined application with PDT consisted of four treatments given 4 days apart, started immediately following photodynamic light delivery, i.e. on days 0, 4, 8 and 12 relative to PDT. The most effective was a combination of intraperitoneal and peritumoral (subtumoral) DBPMAF injections given on each of the treatment days. The rationale for staging multiple DBPMAF injections at 4-day intervals comes from earlier studies (Yamamoto et al, 1988), which demonstrated that the half-life of the activated state of macrophages is about 5–6 days. Since PDT induces inflammation, macrophages activated by the systemic DBPMAF administration are presumably chemotactically recruited to the PDT-treated lesions (Korbelik, 1996). Additionally, peritumoral DBPMAF injection facilitates in situ macrophage activation.

No further benefit was observed with increasing the DBPMAF dose five times higher than that depicted in Figures 1–4; the tumour cure rates, in fact, decreased (data not shown). It appears, therefore, that overexposure to DBPMAF results in macrophage deactivation. This is consistent with our in vitro results showing that macrophage activation was reduced as dosages of this agent increased over the optimum level (N Yamamoto, unpublished results).

DBPMAF and GcMAF can be generated only from the glycosylated DBP. They are highly conserved proteins. The amino acid sequence of mouse DBP is 78% identical to human Gc and 91% identical to rat DBP (Cooke and Haddad, 1989). The DBP protein of all species carries only one oligosaccharide near the C-terminal. Gc1, one of the major Gc isoforms, is 100% glycosylated. In

© Cancer Research Campaign 1997

contrast, only 10% of mouse DBP molecules are glycosylated (Yamamoto and Naraparaju, 1996*a*). As non-glycosylated DBP cannot be converted into DBPMAF, our mouse DBPMAF preparation contained the active form in only 10% of the total protein. Nevertheless, the DBPMAF treatment was, in the present study, more effective in potentiating the anti-tumour effect of PDT than the equivalent GcMAF therapy. Such finding may be explained by the possibility that mice can develop antibodies against the human protein (GcMAF), which would reduce its effectiveness with regimens involving multiple administration of this agent over protracted time intervals. Commercial availability of interspecies antibodies against Gc protein suggests that interspecific antibodies are raised against non-homologous amino acid sequences of DBP. Although the induction of immunosuppression by PDT would have the potential of impairing the production of antibodies to GcMAF in treated mice, this can be abrogated by the reversal of the immunosuppressive effect following treatment with macrophage-activating factor adjuvant to PDT (Figure 6).

In early clinical studies, multiple weekly administrations of GcMAF alone produced excellent responses in a variety of human cancers (N. Yamamoto et al, unpublished results), with no signs of side-effects (Naraparaju et al, 1996). Given as a single modality, this agent appears to be more effective against slow-growing human tumours than against rapid-growing solid mouse tumours. However, non-solid mouse tumours are sensitive to GcMAF therapy. All mice transplanted with $10^5$ Ehrlich ascites tumour cells and administered GcMAF (100 ng per mouse) on days 0 and 4 (or days 4 and 8) after transplantation survived over 65 days, whereas all GcMAF-untreated mice died at $13 \pm 3$ days (Koga et al, 1996).

The mechanism of the potentiation of tumour response to PDT by DBPMAF (GcMAF) features enhanced participation of the host in the eradication of treated cancer. DBPMAF and GcMAF were shown to activate macrophages, monocytes and other phagocytes (oesteoclasts, microglial cells, etc.) (Yamamoto and Naraparaju, 1996*b*), but will not directly stimulate B and T cells (Naraparaju and Yamamoto, 1994). In addition to markedly enhancing the phagocytic and superoxide-generating capacities of macrophages (referred to in the introduction), the macrophage counts dramatically increase following DBPMAF or GcMAF treatment (Yamamoto et al, 1994). This mitogenic effect suggests that the expansion of macrophage populations from progenitors that are not terminally differentiated may also be induced. Therefore, it appears likely that macrophages, activated both systemically and locally by DBPMAF therapy combined with PDT, become more effectively involved in killing cancerous cells, as well as in phagocytosis of tumour cell debris. The fact that an optimized DBPMAF treatment regimen calls for repeated injections extending to 12 days after PDT may reflect the engagement of activated macrophages in the elimination of the islets of tumour cells remaining viable after the PDT treatment. Serving as professional antigen-presenting cells, macrophages can also secure improved processing of tumour antigens and their presentation (in the context of MHC class II molecules) to helper T cells. Through this activity, DBPMAF-activated macrophages would facilitate the development of T-cell-specific tumour immunity (Korbelik, 1996).

Another role of DBPMAF in enhancing the destruction of PDT-treated tumours is suggested by the observation that the immunopotentiation by DBPMAF overwhelmed the PDT-induced immunosuppression (as demonstrated by the DTH response). In accordance with the findings of other investigators (Elmets et al, 1986; Lynch et al, 1989), the treatment of SCCVII tumours by PDT resulted in a markedly diminished DTH response in the host animals. By blocking this immunosuppressive effect, the DBPMAF therapy may have permitted a more pervasive tumour destruction by PDT (Korbelik, 1996). Since macrophage activation for phagocytosis and subsequent antigen presentation is the first step of the immune development process, the PDT-induced immunosuppression was suggested to be related to the impairment of macrophage activation by PDT (Yamamoto et al, 1992). The adjuvant administration of DBPMAF bypasses the decapitated macrophage activation cascade (Yamamoto et al, 1994) leading to reversal of the immunosuppressive state.

The observed synergism of PDT combined with DBPMAF treatment provides direct evidence that PDT is highly receptive to the adjuvant treatment with a macrophage-activating factor. This is consistent with our earlier findings with the SCCVII carcinoma model, which showed that the PDT response of this poorly immunogenic tumour is enhanced by a combined treatment with glucan SPG (Krosl and Korbelik, 1994) and cytokine granulocyte–macrophage colony-stimulating factor (GM-CSF) (Krosl et al, 1996); both these agents stimulate macrophage activity. On the other hand, it was reported that the response of immunogenic murine tumours to PDT is augmented by the adjuvant treatment with BCG (Cho et al, 1992), *Corynebacterium parvum* vaccine (Myers et al, 1989) or mycobacterial cell wall extract (Korbelik and Krosl, 1996), which are also immune stimulants acting primarily on macrophages. Together, these results raise the possibility that PDT with adjuvant macrophage-directed immunotherapy may eventually prove clinically useful for achieving improved control of solid cancers.

### ABBREVIATIONS

DBP, vitamin $D_3$-binding protein; DBPMAF, vitamin $D_3$-binding protein-derived macrophage-activating factor; DNFB, 2,4-dinitrofluorobenzene; DTH, delayed-type contact hypersensitivity; GcMAF, Gc protein-derived macrophage-activating factor; i.p., intraperitoneally; i.v., intravenously; mDBP, mouse DBP; PDT, photodynamic therapy; p.t., peritumorally.

### ACKNOWLEDGEMENTS

The technical assistance provided by Sandy Lynde is gratefully acknowledged. This research was supported by Grant MT-12165 from the Medical Research Council of Canada and US Public Health Service Grant AI-32140.

### REFERENCES

Cho Y-H, Straight RC and Smith JH (1992) Effects of photodynamic therapy in combination with intravesical drugs in a murine bladder tumor model. *J Urol* **147**: 743–746

Cooke NE and Haddad JG (1986) Vitamin D binding protein (Gc-globulin). *Endocrine Rev* **10**: 294–307

Elmets CA and Bowen KD (1986) Immunological suppression in mice treated with hematoporphyrin derivative photoradiation. *Cancer Res* **46**: 1608–1611

Evans S, Matthews W, Perry R, Fraker D, Norton J and Pass HI (1990) Effect of photodynamic therapy on tumor necrosis factor production by murine macrophages. *J Natl Cancer Inst* **82**: 34–39

Fisher AMR, Murphree AL and Gomer CJ (1995) Clinical and preclinical photodynamic therapy. *Lasers Surg Med* **17**: 2–31

Koga Y, Naraparaju VR and Yamamoto N (1996) Antitumor effects of vitamin $D_3$-binding protein-derived macrophage activating factor on Ehrlich tumor bearing mice. *87th Ann Am Assoc Cancer Res Proc* **37**: 481

© Cancer Research Campaign 1997

Korbelik M (1996) Induction of tumor immunity by photodynamic therapy. *J Clin Laser Med Surg* **17**: 329–334

Korbelik M and Krosl G (1994) Enhanced macrophage cytotoxicity against tumor cells treated with photodynamic therapy. *Photochem Photobiol* **60**: 497–502

Korbelik M and Krosl G (1996) Photosensitizer distribution and photosensitized damage of tumor tissues. In *The Fundamental Bases of Phototherapy*, Hönigsmann H, Jori G and Young AR (eds), pp. 229–245. OEMF spa: Milan

Korbelik M, Krosl G, Naraparaju VR and Yamamoto N (1995) The effect of enzymatically generated macrophage activating factor (GcMAF) on the tumor response to photodynamic therapy. *Photochem Photobiol* **61**: 97S

Korbelik M, Krosl G, Krosl J and Dougherty GJ (1996) Modulation of tumor response to photodynamic therapy in severe combined immunodeficient (SCID) mice by adoptively transferred lymphoid cells. *SPIE* **2675**: 156–162

Krosl G and Korbelik M (1994) Potentiation of photodynamic therapy by immunotherapy: the effect of schizophyllan (SPG). *Cancer Lett* **84**: 43–49

Krosl G, Korbelik M and Dougherty GJ (1995) Induction of immune cell infiltration into murine SCCVII tumour by Photofrin-based photodynamic therapy. *Br J Cancer* **71**: 549–555

Krosl G, Korbelik M, Krosl J and Dougherty GJ (1996) Potentiation of photodynamic therapy elicited antitumor response by localized treatment with granulocyte–macrophage colony stimulating factor. *Cancer Res* **56**: 3281–3286

Link RP, Perlman KL, Pierce EA, Schnoes HK and Deluca HF (1986) Purification of human serum vitamin D-binding protein by 25-hydroxyvitamin $D_3$-Sepharose chromatography. *Anal Biochem* **157**: 262–269

Lynch DH, Haddad S, Vernon KJ, OTT MJ, Straight RC and Jolles CJ (1989) Systemic immunosuppression induced by photodynamic therapy (PDT) is adoptively transferred by macrophages. *Photochem Photobiol* **49**: 453–458

Myers RC, LAU BHS, Kunihira DY, Torrey RR, Woolley JL and Tosk J (1989) Modulation of hematoporphyrin derivative-sensitized phototherapy with *Corynebacterium parvum* in murine transitional cell carcinoma. *Urology* **33**: 230–235

Naraparaju VR and Yamamoto N (1994) Roles of β-galactosidase of B lymphocytes and sialidase of T lymphocytes in inflammation-primed activation of macrophages. *Immunol Lett* **43**: 143–148

Naraparaju VR, Wimmers RS, Neil RN, Orchard PJ and Yamamoto N (1996) Origin of immunosuppression in juvenile leukemia and therapeutic efficacy of vitamin $D_3$-binding protein-derived macrophage activating factor. *87th Ann Am Assoc Cancer Res Proc* **37**: 213

Suit HD, Sedlacek, RS, Silver G and Dosoretz D (1985) Pentobarbital anesthesia and the response of tumor and normal tissue in the C3Hf/Sed mouse to radiation. *Radiat Res* **104**: 47–65

Yamamoto N and Homma S (1991) Vitamin $D_3$ binding protein (group-specific component) is a precursor for the macrophage activating signal factor from lysophospahtidylcholine-treated lymphocytes. *Proc Natl Acad Sci USA* **88**: 8539–8543

Yamamoto N and Naraparaju VR (1996*a*) Role of mouse vitamin $D_3$-binding protein in activation of macrophages. *J Immunol* **157**: 1744–1751

Yamamoto N and Naraparaju VR (1996*b*) A defect in inducible β-galactosidase of B lymphocytes in the osteopetrotic (*mi-my*) mouse. *Immunology* **88**: 605–611

Yamamoto N, St Claire DA, Homma S and Ngwenya BZ (1988) Activation of mouse macrophages by alkylglycerols, inflammation products of cancerous tissues. *Cancer Res* **48**: 6044–6049

Yamamoto N, Homma S, Sery TW, Donoso LA and Hoober KJ (1991) Photodynamic immunopotentiation: *in vitro* activation of macrophages by treatment of peritoneal cells with haematoporphyrin derivative and light. *Eur J Cancer* **27**: 467–471

Yamamoto N, Hoober K, Yamamoto N and Yamamoto S (1992) Tumoricidal capacities of macrophages photodynamically activated with hematoporphyrin derivative. *Photochem Photobiol* **56**: 245–250

Yamamoto N, Lindsay DD, Naraparaju VR, Ireland RA and Popoff SN (1994) A defect in the inflammation-primed macrophage-activation cascade in osteopetrotic rats. *J Immunol* **152**: 5100–5107

© Cancer Research Campaign 1997